# Association between the *CD24* Ala57Val polymorphism and risk for multiple sclerosis and systemic lupus erythematosus: a meta-analysis

**DOI:** 10.1038/srep09557

**Published:** 2015-04-01

**Authors:** Jian Huang, Yaqi Yang, Zibin Liang, Miaomiao Kang, Ying Kuang, Feng Li

**Affiliations:** 1 Department of Anatomy and Neurobiology, Zhongshan School of Medicine, Sun Yat-sen University, Guangzhou, China

## Abstract

The cluster of differentiation 24 (*CD24*) Ala57Val polymorphism has been implicated as a risk factor for multiple sclerosis (MS) and systemic lupus erythematosus (SLE); however, genetic studies have produced controversial results. A meta-analysis was performed on this topic. We used odds ratio (OR) and 95% confidence interval (95% CI) to investigate the strength of association. Eleven studies from nine publications consisting of 2466 cases and 2650 controls were included. The results suggested that the *CD24* Val/Val genotypes were associated with an increased risk of MS in all study subjects and Caucasians (OR = 2.28, 95% CI: 1.68–3.10, *P_z_* < 0.001 and OR = 2.30, 95% CI: 1.66–3.20, *P_z_* < 0.001, respectively). Sensitivity analysis showed that no individual study was found to be significantly biasing the pooled results. Although meta-analysis also suggested an association between the *CD24* Val/Val genotypes and SLE risk in Caucasians (OR = 1.71, 95% CI: 1.31–2.24, *P_z_* < 0.001), sensitivity analysis demonstrated that the association was not statistically significant after removing a Spanish study. In conclusion, this meta-analysis suggests that the *CD24* Ala57Val polymorphism is associated with an increased risk of MS in Caucasians. However, the available evidence is not sufficient to support an association between the *CD24* Ala57Val polymorphism and SLE risk.

Multiple sclerosis (MS) and systemic lupus erythematosus (SLE) are autoimmune diseases mediated by self-reactive T cells and other cells of the adaptive and innate immune systems^1^,^2^. MS is characterized by chronic inflammation, multifocal demyelination and axon loss that affect the central nervous system (CNS), whereas in SLE, inflammation and tissue damage can involve many organs and systems, including the skin, lungs, kidneys, and CNS. Although the etiology of these diseases remains largely unknown, it is apparent that both genetic and environmental factors play a role^3^. Human genetic studies have shown that the human leukocyte antigen (*HLA*) gene on chromosome 6p21 is the most important genetic factor for MS and SLE^3^. In MS, the *HLA-DR15* haplotype (DRB1*1501-DQA1*0102-DQB1*0602) and its individual alleles appear to have the main role in Caucasians^4^. In SLE, the *HLA-DR2* and *DR3* alleles and the Class III variants are important genetic susceptibility factors for the disease^5^. However, the HLA complex does not fully explain the genetic susceptibility in these diseases. Recent advances in molecular genetic technologies have led to identification of many single nucleotide polymorphisms (SNPs) outside the HLA region for MS and SLE^6^,^7^. The exploration of non-HLA genetic risk loci may help elucidate signaling pathways involved in the pathogenesis of MS and SLE, and provide insight into genes and mechanisms shared among autoimmune diseases.

Cluster of differentiation 24 (CD24) is a small cell surface protein molecule anchored by glycosyl-phosphotidyl-inositol (GPI) in a wide variety of cell types, including T cells, B cells, dendritic cells (DCs), cancer cells and local antigen-presenting cells in the CNS^8^,^9^,^10^,^11^,^12^. CD24 has been implicated in a CD28-independent costimulatory pathway in the activation of CD4+ and CD8+ T cells^13^. It also functions as an important regulator during the early stages of B and T cell lymphopoiesis^14^. In addition, CD24 modulates the interaction between very late antigen (VLA)-4 and vascular cell adhesion molecule (VCAM)-1^15^. These adhesion molecules play an important role in lymphocyte costimulation and migration to sites of inflammation. The human *CD24* gene, which maps on 6q21, is considered a candidate gene for MS and SLE. A C > T single-nucleotide polymorphism (SNP) is located in the presumable GPI-anchor cleavage site of the CD24 protein (rs52812045), leading to the replacement of an alanine (*CD24* Ala) by a valine (*CD24* Val) (Ala57Val)^16^. According to the data provided by the National Center for Biotechnology Information Entrez SNP, the Val allele frequency is 0.320 in a population from Centre d'Etude du Polymorphisme Human pedigrees. Several studies have investigated the relationship of the *CD24* Ala57Val polymorphism with MS and SLE^17^,^18^,^19^,^20^,^21^,^22^,^23^,^24^,^25^; however, results from individual studies are not consistent. This likely stems from several facors, including underpowered sample sizes, ethnic differences and minor genetic effects. We therefore performed a meta-analysis of published case-control studies to evaluate the association between the *CD24* Ala57Val polymorphism and risk for MS and SLE.

## Methods

### Search strategy and identification of studies

An electronic search of the medical literature was conducted to identify genetic association studies assessing the relationship of the *CD24* Ala57Val polymorphism with MS and SLE. PubMed, Scopus, Web of Knowledge, China National Knowledge Infrastructure (CNKI) and Wanfang databases were searched for papers published until January 2015 without language restrictions. Search terms included “multiple sclerosis”, “MS”, “systemic lupus erythematosus”, “SLE”, “cluster of differentiation 24”, “CD24”, “genetics”, “polymorphism”, “SNP”, “rs52812045”, and “association”. The reference lists of retrieved publications were also reviewed to identify other relevant studies missed by the database search. The following inclusion criteria were used for study selection: (1) case-control design; (2) genotype frequency data for both cases and controls were available; (3) literature published in English or Chinese. Studies were excluded if they met the following criteria: (1) studies on animal populations; (2) no control subjects; (3) duplicate data; (4) insufficient data. Reviewers screened study reports by first screening titles and abstracts to select publications for full-text evaluation, then screening full-text publications to confirm eligibility of the studies.

### Data extraction

Data were extracted by two of the authors, and differences were resolved by consensus. For each study, the following data were extracted: first author name, publication year, country, ethnicity, disease, sample size, genotypic frequencies of the CD24 Ala57Val SNP, diagnostic criteria and genotyping method. We assumed study subjects to be Caucasians, if Caucasians comprised 90% of the subjects. When a publication reported results on different subpopulations according to country, each subpopulation was considered as a separate study in this meta-analysis.

### Methodological quality assessment

The quality of selected studies was assessed by scoring according to the Newcastle Ottawa Scale (NOS) (www.ohri.ca/programs/clinical_epidemiology/oxford.asp), awarding points based on selection of cases and controls, comparability of cases and controls, and ascertainment of exposure (Supplementary Table 1). A maximum of 9 points could be awarded, where 6 or more points was considered high quality.

### Statistical analyses

Meta-analysis was conducted using STATA version 11.0. Odds ratios (ORs) with 95% confidence intervals (CI) were used to evaluate the size and strength of relationship of the CD24 Ala57Val polymorphism with MS and SLE. ORs were calculated from genotype frequency data without adjustment. Z-test was used for assessing the significance of the pooled OR, with *P* < 0.05 considered statistically significant. Three pooled ORs were calculated: Val/Val versus Ala/Ala (OR1), Ala/Val versus Ala/Ala (OR2), and Val/Val versus Ala/Val (OR3). The resulting ORs were used to determine the most appropriate genetic model using a previously described approach^26^:

- If OR1 = OR3 ≠ 1 and OR2 = 1, a recessive model is suggested;
- If OR1 = OR2 ≠ 1 and OR3 = 1, a dominant model is suggested;
- If OR2 = 1/OR3 ≠ 1 and OR1 = 1, an over dominant model is suggested;
- If OR1 > OR2 > 1 and OR1 > OR3 > 1 (or OR1 < OR2 < 1 and OR1 < OR3 < 1), a co-dominant model is suggested;

Heterogeneity was assessed by Cochrane's Q test and the I-square statistic. A *P*-value of <0.10 for the Q test was considered to indicate heterogeneity across studies, in which case the DerSimonian Laird random-effects model was fitted to calculate the pooled OR^27^. Otherwise, the standard Mantel-Haenszel fixed-effects model was fitted^28^. A cumulative meta-analysis was also conducted to demonstrate how evidence concerning the genetic association has evolved over time. Sensitivity analysis was performed to determine whether the results were considerably influenced by any single study. Funnel plots were created to assess publication bias by plotting natural logarithm of individual study effect size against the standard error of the natural logarithm of individual study effect size. Begg's test and Egger's test were also used to evaluate publication bias, with *P* < 0.05 being considered statistically significant. The pooled frequency of the Val allele in control groups from Caucasian studies was calculated using Meta-Analyst version 3.13. A power calculation was carried out using Power and Sample Size Calculation version 3.1.2 (http://biostat.mc.vanderbilt.edu/twiki/bin/view/Main/PowerSampleSize). To assess whether the genotype frequencies in control groups were in Hardy-Weinberg equilibrium (HWE) we used a web-based programme (http://www.oege.org/software/hwe-mr-calc.html).

## Results

### Characteristics of published studies

**Figure 1:**
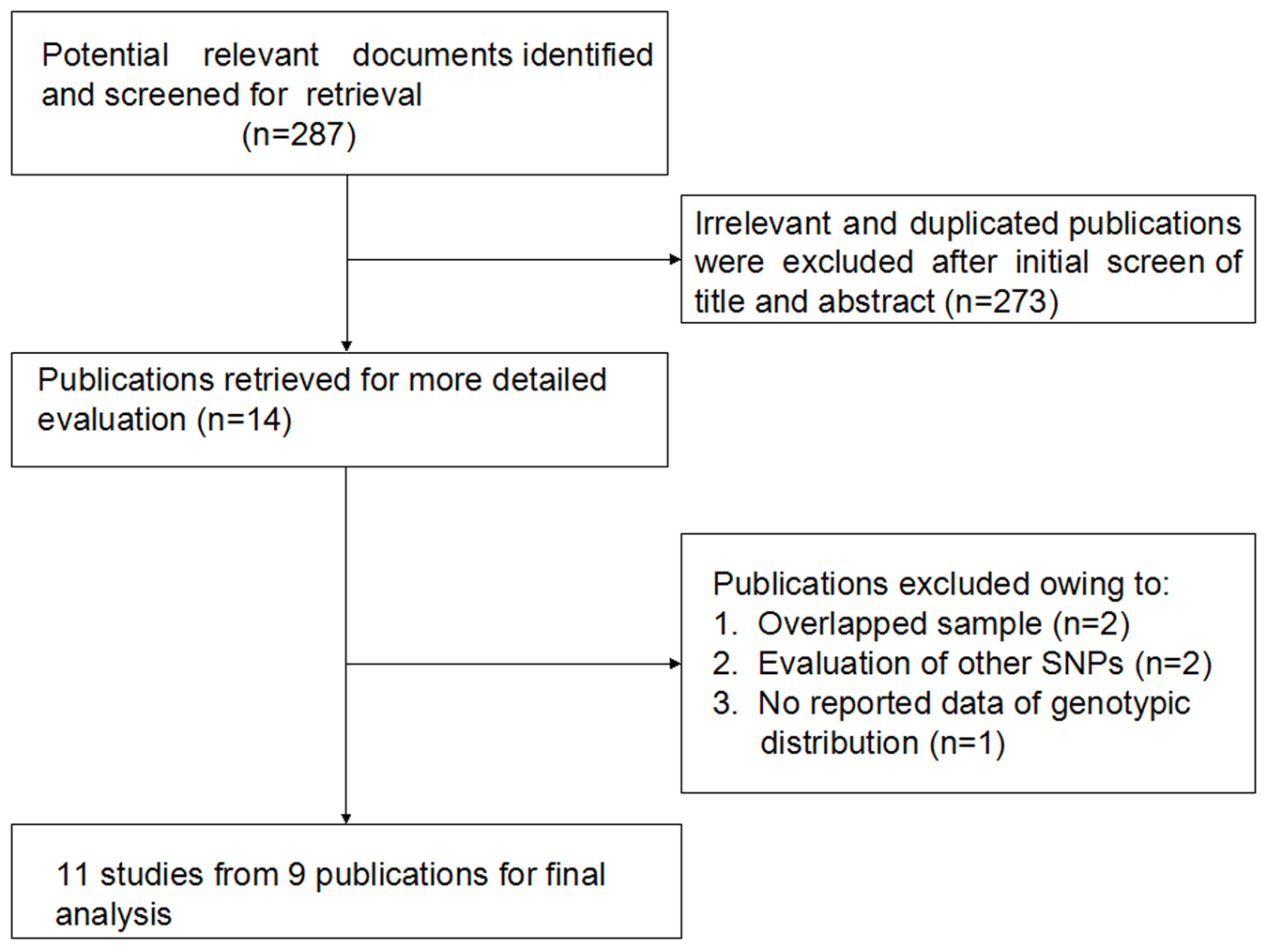
Flow diagram of studies included in the meta-analysis.

A flow diagram showing the selection process of studies included in the present analysis is shown in Fig. 1. After title and abstract evaluation, 273 publications were excluded either because they were irrelevant, not about human subjects, or the record was a duplicate search result. Five publications were excluded after evaluating the remaining 14 publications in their entirety. Overall, 11 studies from nine publications met our inclusion criteria with a total of 2466 cases and 2650 controls^17^,^18^,^19^,^20^,^21^,^22^,^23^,^24^,^25^. In terms of disease, six studies with 899 cases and 1127 controls evaluated the association between the *CD24* Ala57Val polymorphism and MS risk^17^,^18^,^19^,^21^,^23^,^24^, whereas five studies with 1567 cases and 1523 controls from three publications assessed the relationship of this SNP with SLE^20^,^22^,^25^. It was noteworthy that the study by Sánchez et al. included three cohorts: a Spanish cohort, a German cohort and a Swedish cohort^20^. In terms of ethnicity, nine studies from seven publications were undertaken in Cancasians^17^,^19^,^20^,^21^,^22^,^23^,^24^, whereas two studies were conducted in Asians^18^,^25^. The Val allele frequency in controls ranged from 23.8% to 31.0% among Caucasian studies, and the pooled frequency was 27.6 (95% CI: 25.7–29.5). Table 1 summarizes the characteristics of the eligible studies included in this meta-analysis.

### Association between the *CD24* Ala57Val polymorphism and MS

**Figure 2:**
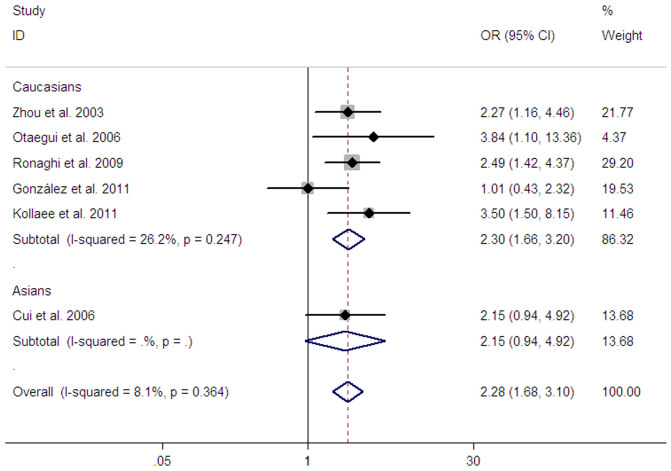
Meta-analysis of the association between the *CD24* Ala57Val polymorphism and MS risk in recessive model.

For this polymorphism, five studies containing 816 cases and 1017 controls were undertaken in Caucasians, whereas one study with 83 cases and 110 controls were conducted in Asians. Genotype-specific odds ratios OR1, OR2, and OR3 were 2.47 (95% CI: 1.79–3.40, *P_z_* < 0.001), 1.17 (95% CI: 0.97–1.42, *P_z_* = 0.104), and 2.09 (95% CI: 1.51–2.90, *P_z_* < 0.001), respectively. These genotype-specific ORs were most suggestive of a recessive model (Val/Val vs Ala/Val + Ala/Ala). In this model, meta-analysis demonstrated a significant association between the *CD24* Ala57Val polymorphism and MS risk in all study subjects (OR = 2.28, 95% CI: 1.68–3.10, *P_z_* < 0.001) (Table 2 and Fig. 2). Subgroup analysis according to ethnicity demonstrated a significant association between the *CD24* Ala57Val polymorphism and MS risk in Caucasians in recessive model (OR = 2.30, 95% CI: 1.66–3.20, *P_z_* < 0.001) (Table 2 and Fig. 2). Ethnicity-specific analysis could not be conducted in Asians because there was only one eligible study. Of note is that two Caucasian studies presented deviation from HWE^19^,^21^. Omitting these studies did not change the results of the pooled analyses for all study subjects and Caucasians (OR = 2.09, 95% CI: 1.42–3.06, *P_z_* < 0.001 and OR = 2.07, 95% CI: 1.35–3.19, *P_z_* = 0.001, respectively). Sensitivity analysis was conducted to confirm the stability and liability of the meta-analysis by removing each of the involved study in turn. No individual study was found to be significantly biasing the pooled results (data not shown). A cumulative meta-analysis based on the publication date showed a significant and consistent trend toward increased risk of MS for the Val/Val genotypes over time and the trend stabilized by 2009 (Supplementary Fig. 1). Heterogeneity among the pooled ORs for all study subjects and Caucasians was low (*P* = 0.364, I^2^ = 8.1 and *P* = 0.247, I^2^ = 26.2, respectively) (Table 2 and Fig. 2). When we further stratified studies that used Caucasian samples according to diagnostic criteria (McDonald criteria^29^ or Poser criteria^30^), the I^2^ value for heterogeneity decreased from 26.2% to 0%.

### Association between the *CD24* Ala57Val polymorphism and SLE

**Figure 3:**
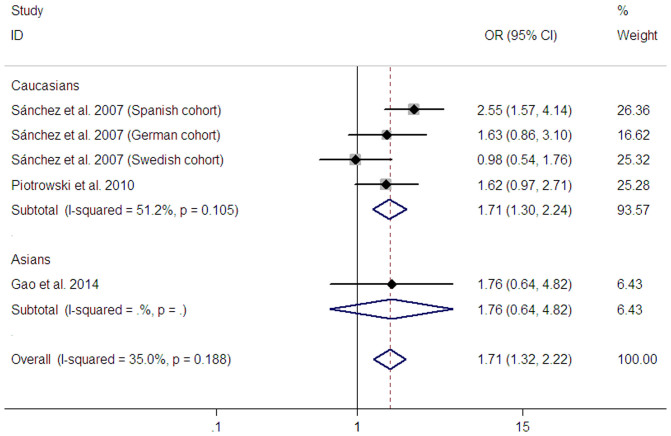
Meta-analysis of the association between the *CD24* Ala57Val polymorphism and SLE risk in recessive model.

Five studies from three publications assessed the polymorphism. Among them, four studies were conducted in Caucasians, whereas one study was performed in Asians. Of note is that all studies presented no deviation from HWE. Genotype-specific odds ratios OR1, OR2, and OR3 were 1.90 (95% CI: 1.45–2.50, *P_z_* < 0.001), 1.17 (95% CI: 1.01–1.36, *P_z_* = 0.039), and 1.54 (95%CI: 1.17–2.02, *P_z_* = 0.002), respectively. These estimates suggested a recessive effect of the T allele (*CD24* Val) for SLE. Therefore, Val/Val was compared with Ala/Val and Ala/Ala genotypes combined. In this model, a significant association between the *CD24* Ala57Val polymorphism and SLE risk was found in all study subjects (OR = 1.71, 95% CI: 1.32–2.22, *P_z_* < 0.001) (Table 2 and Fig. 3). A cumulative meta-analysis based on publication year showed that 95% CIs progressively became narrower over time (Supplementary Fig. 2). Subgroup analysis by ethnicity indicated an association between the *CD24* polymorphism and SLE risk in Caucasians (OR = 1.71, 95% CI: 1.31–2.24, *P_z_* < 0.001) (Table 2 and Fig. 3). However, sensitivity analysis showed that the association in Caucasians became statistically non-significant after removing a spanish cohort^20^ (data not shown). We did not perform subgroup analysis in Asians in that there was only one Asian study^25^. Heterogeneity among the pooled ORs for all study subjects and Caucasians was moderate (*P* = 0.188, I^2^ = 35.0% and *P* = 0.105, I^2^ = 51.2%, respectively). We further performed subgroup analysis in Caucasian studies according to sample size (subjects **≥** 1000 or subjects < 1000) and genotyping method. In subgroup analysis based on sample size, the I^2^ value for heterogeneity decreased from 51.2% to 0% (Table 2), but it did not significantly decrease in subgroup analysis by genotyping method (not shown).

### Publication bias

**Figure 4:**
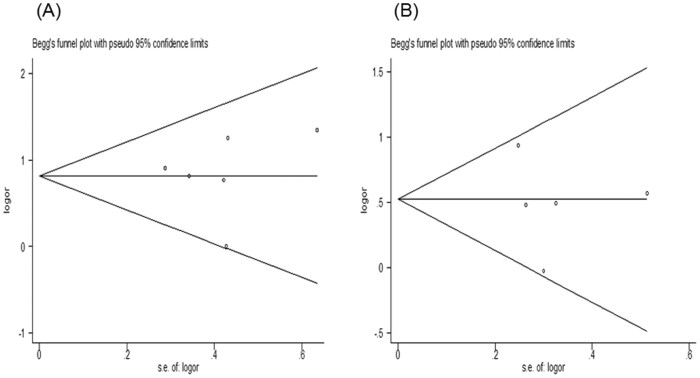
(A): Begg's funnel plot for the *CD24* Ala57Val polymorphism and MS risk in recessive model; (B): Begg's funnel plot for the *CD24* Ala57Val polymorphism and SLE risk in recessive model.

Funnel plots offer a visual sense of the relationship between effect size and precision for publication bias amongst the studies in meta-analysis. Figure 4 showed the funnel plots of meta-analyses evaluating the relationship of the *CD24* Ala57Val polymorphism with MS and SLE in recessive model (Fig. 4A for MS and Fig. 4B for SLE). The shape of each funnel plot seemed symmetrical. The results of Begg's test and Egger's test suggested no evidence for publication bias (Table 3).

## Discussion

It is generally believed that variants of immune-related genes modulate genetic predisposition to autoimmune diseases, including MS and SLE. The *CD24* Ala57Val polymorphism has been implicated in the susceptibility to MS and SLE and has been assessed in many association studies. However, the outcomes of these studies remain contradictory rather than convincing. The inconsistency seems to be mainly owing to the heterogeneity of the populations under study and the small sample size of most studies, leading to little statistical power. Meta-analysis is a quantitative statistical analysis that integrates the results of several independent studies in order to see whether the overall effect is significant. It has been increasingly used in evaluating the association between predisposing genes and complex traits, including MS, SLE, type 1 diabetes and inflammatory bowel disease^32^. In the present study, we undertook a meta-analysis to investigate the relationship of the *CD24* Ala57Val polymorphism with MS and SLE. The results indicated that the *CD24* Val/Val genotypes were associated with increased risk of MS in Caucasians, whereas current evidence is still insufficient to suggest an association between the *CD24* Val/Val genotypes and SLE risk.

Among eligible studies evaluating the *CD24* Ala57Val polymorphism and MS, two studies presented deviation from HWE. After exclusion of them, the association in all study subjects and Caucasians remained statistically significant. In addition, sensitivity analysis and cumulative meta-analysis further strengthened the validity of the results. Thus, the association of the *CD24* Val/Val genotypes with MS revealed by this meta-analysis can be considered reliable. Although meta-analysis also showed an association between the polymorphism and SLE in Caucasians, sensitivity analysis demonstrated that the association was not statistically significant after removing a Spanish cohort^20^. The exclusion of even one study can lead to the loss of statistical significance, indicating that the result for SLE is not stable. It is necessary to further evaluate the *CD24* Ala57Val polymorphism and SLE in additional ethnic groups to achieve a definitive conclusion.

In this meta-analysis, low or moderate heterogeneity was identified. Subgroup analyses indicated that diagnostic criteria was the major factor for heterogeneity among Caucasian studies evaluating the polymorphism and MS, whereas sample size was largely responsible for the heterogeneity of results of SLE.

CD24 is expressed in various immune cell types and is known to mediate several important functions. Previous work has established that CD24 functions as a costimulatory regulator for T cell clonal expansion. CD24 expression on T cells is required for the homeostatic proliferation of both CD4 and CD8 T cells in the lymphopenic environment^33^. Besides T cells, CD24 participates in the activation and differentiation of B cells. *CD24*-deficiency leads to a reduction in late pre-B and immature B-cell populations in the bone marrow, suggesting that CD24 expression influences the maturation of B cells^9^. In addition, CD24 can bind to P-selection and regulate VLA-4 binding to either VCAM-1 or fibronectin^15^, which may contribute to recruitment of leukocytes to inflamed tissues. Moreover, it has been reported that CD24 mediates intracellular signaling via a glycolipid-enriched membrane (GEM) -dependent mechanism^34^. The role of CD24 in autoimmunity has not been clearly delineated. Bai et al. previously reported that mice with a targeted mutation of CD24 were highly resistant to experimental autoimmune encephalomyelitis (EAE) induced by immunization with myelin oligodendrocyte glycoprotein (MOG)-peptide^35^. In addition, it was observed that mice with overexpressed CD24 in the CNS developed severe EAE^36^, whose development requires CD24 expression on both T cells and non-T host cells. CD24 functions as an important regulator for local clonal expansion and persistence of T cells after their migration into the CNS during the development of EAE^10^. It is known that thymic clonal deletion of autoantigen reactive T cells is important for self-tolerance. Joseph and coworkers found that mice null for CD24 exhibited much more efficient clonal deletion than wild-type mice, suggesting that CD24 actively suppressed clonal deletion and was a critical determinant in autoimmunity^37^. Apart from EAE, CD24 may also contribute to the pathogenesis of experimental SLE. The New Zealand black (NZB) mice and their F1 cross with the New Zealand white mice (NZB/W) displays a lupus-like autoimmune disease and are a well-known model of human SLE. It was reported that up-regulation of CD24 and other co-stimulatory molecules were linked to polyclonal B cell activation in NZB and NZB/W mice, playing a critical role in the loss of tolerance and production of pathogenic autoantibodies^38^. These lines of evidence suggest that the *CD24* gene may be a candidate gene for MS and SLE. It has been speculated that the *CD24* Ala57Val SNP leads to a nonconservative replacement from alanine to valine at the site which immediately precedes the putative cleavage site for GPI anchor^17^. Since the alanine and valine are substantially different in size, the replacement may increase the efficiency of cleavage and result in an increased expression of CD24 in the *CD24* Val/Val genotypes. Zhou et al. reported that *CD24* Val allele lead to 30–40% more cell surface expression of CD24 than the *CD24* Ala allele^17^. The enhanced induction of CD24 may be an important checkpoint for the pathogenesis of MS and SLE. In addition, it was reported that the *CD24* Val allele was associated with the production of autoantibodies in patients with SLE^22^. To clarify the role of the *CD24* SNP in MS and SLE, more functional assays are needed in the future.

The present meta-analysis has some limitations that should be acknowledged. First, the key threat to any meta-analysis is that of reporting bias. Although there is no evidence of publication bias (Table 3 and Fig. 4), it is not possible to rule it out entirely. Some findings of no association between the *CD24* Ala57Val polymorphism and risk of MS and SLE may not have been reported in the literature and therefore could not be included in our meta-analysis. Second, the results of our meta-analysis were based on the data from case-control studies, because they were most frequently used in association studies to evaluate the *CD24* Ala57Val polymorphism. We expect that as more studies of other types, such as prospective cohort and family-based studies become available, a more comprehensive systematic review and meta-analysis can be conducted to provide additional information on the role of this SNP in MS and SLE. Third, we only evaluated the *CD24* Ala57Val polymorphism in this meta-analysis. Because of the limitation of the published data, we were unable to assess the relationship of other *CD24* polymorphisms, such as the −809 C/A, −534A/C and −492G/C polymorphisms in the promoter region with MS and SLE. There were insufficient data to evaluate linkage disequilibrium between the *CD24* Ala57Val SNP and other polymorphisms. Fourth, because most eligible studies were conducted in Caucasians, the results of this meta-analysis were mainly applicable to this ethnic group. Subgroup analysis could not be performed in Asians due to insufficient data. Future studies should be conducted in other ethnic groups besides Caucasians.

In conclusion, the present meta-analysis of the literature reveals that the Val/Val genotypes of the *CD24* Ala57Val polymorphism are associated with increased risk of MS in Caucasians. However, the available evidence is not sufficient to support an association between the *CD24* Ala57Val polymorphism and SLE risk.

## Supplementary Material

Supplementary Information Supplementary Information

## Figures and Tables

**Table 1:** Characteristics of eligible studies in meta-analysis

Author [Ref]	Country	Ethnicity	Disease	Diagnostic criteria	Number		Cases			Controls			Val allele frequency in controls (%)	Genotyping method	Score	HWE	Power (%)
					Cases	Controls	Ala/Ala	Ala/Val	Val/Val	Ala/Ala	Ala/Val	Val/Val					
Zhou et al [17]	USA	Caucasians	MS	McDonald criteria^29^	242	207	113	97	32	109	85	13	26.8	PCR-RFLP	7	Yes	69.0
Cui et al [18]	China	Asians	MS	Poser criteria^30^	83	110	25	42	16	48	51	11	33.2	PCR-RFLP	7	Yes	45.3
Otaegui et al [19]	Spain	Caucasians	MS	McDonald criteria	135	285	59	69	7	145	136	4	25.3	PCR-RFLP	8	No	60.0
Sánchez et al [20]	Spain, Sweden, Germany	Caucasians	SLE	American College of Rheumatology criteria^31^	696 (Spanish)	539 (Spanish)	356	269	71	305	211	23	23.8	TaqMan allele discrimination assay	8	Yes	98.0
					257 (German)	317 (German)	129	105	23	161	138	18	27.4		8	Yes	33.1
					310 (Swedish)	247 (Swedish)	141	142	27	117	108	22	30.8		8	Yes	5.1
Ronaghi et al [21]	Iran	Caucasians	MS	McDonald criteria	217	200	102	68	47	114	66	20	26.5	PCR-RFLP	5	No	90.5
Piotrowski et al [22]	Poland	Caucasians	SLE	American College of Rheumatology criteria	250	350	91	125	34	166	153	31	30.7	PCR-RFLP	8	Yes	45.5
González et al [23]	Argentina	Caucasians	MS	Poser criteria	102	205	43	50	9	96	91	18	31.0	PCR-RFLP	7	Yes	5.0
Kollaee et al [24]	Iran	Caucasians	MS	McDonald criteria	120	120	56	40	24	63	49	8	27.1	PCR-RFLP	8	Yes	86.4
Gao et al [25]	China	Asians	SLE	American College of Rheumatology criteria	54	70	16	28	10	40	22	8	27.1	PCR-RFLP	7	Yes	30.5

HWE, Hardy-Weinberg equilibrium; MS, multiple sclerosis; PCR-RFLP, polymerase chain reaction-restriction fragment length polymorphism; SLE, systemic lupus erythematosus; USA, united states of America.

**Table 2:** Pooled odds ratios for recessive model for the *CD24* Ala57Val polymorphism

Disease	Variables	No. of studies	Test of association		Test of heterogeneity	
			OR (95% CI)	*P_z_*	*P_het_*	I^2^
MS	Total	6	2.28 (1.68–3.10)	<0.001	0.364	8.1%
	Subgroup analysis by HWE status					
	Deviation from HWE	2	2.66 (1.59–4.46)	<0.001	0.533	0.0%
	No deviation from HWE	4	2.09 (1.42–3.06)	<0.001	0.219	32.2%
	Subgroup analysis by ethnicity					
	Caucasians	5	2.30 (1.66–3.20)	<0.001	0.247	26.2%
	Poser criteria^a^	1	1.01 (0.44–2.32)	0.990	NA	NA
	McDonald criteria^b^	4	2.68 (1.86–3.87)	<0.001	0.801	0.0%
SLE	Total	5	1.71 (1.32–2.22)	<0.001	0.188	35.0%
	Subgroup analysis by ethnicity					
	Caucasians	4	1.71 (1.31–2.24)	<0.001	0.105	51.2%
	Subjects ≥ 1000^c^	1	2.55 (1.57–4.14)	<0.001	NA	NA
	Subjects < 1000^d^	3	1.38 (1.00–1.92)	0.057	0.375	0.0%

CI, confidence interval; HWE, Hardy-Weinberg equilibrium; MS, multiple sclerosis; NA, not available; OR, odds ratio; *P_het_*, *P*-value for heterogeneity; *P_z_*, *P*-value for overall effect; SLE, systemic lupus erythematosus.

aCaucasian studies which used Poser criteria.

bCaucasian studies which used McDonald criteria.

cCaucasian studies which included no less than 1000 subjects.

dCaucasian studies which included less than 1000 subjects.

**Table 3:** Begg's test and Egger's test for evaluating publication bias

Disease	Population	*P* value of Begg's test	*P* value of Egger's test
MS	Overall	1.000	0.857
	Caucasians	0.806	0.861
SLE	Overall	1.000	0.677
	Caucasians	0.734	0.340

MS, multiple sclerosis, SLE, systemic lupus erythematosus.

## References

[b1] Azevedo P. C., Murphy G. & Isenberg D. A. Pathology of systemic lupus erythematosus: the challenges ahead. Methods Mol Biol. 1134, 1–16 (2014). 24497350 10.1007/978-1-4939-0326-9_1

[b2] Ciccarelli O. *et al.* Pathogenesis of multiple sclerosis: insights from molecular and metabolic imaging. Lancet Neurol. 13, 807–822 (2014). 25008549 10.1016/S1474-4422(14)70101-2

[b3] Lettre G. & Rioux J. D. Autoimmune diseases: insights from genome-wide association studies. Hum Mol Genet. 17, R116–121 (2008). 18852199 10.1093/hmg/ddn246 PMC2782355

[b4] Allen M. *et al.* Association of susceptibility to multiple sclerosis in Sweden with HLA class II DRB1 and DQB1 alleles. Hum Immunol. 39, 41–48 (1994). 8181961 10.1016/0198-8859(94)90099-x

[b5] Deng Y. & Tsao B. P. Genetic susceptibility to systemic lupus erythematosus in the genomic era. Nat Rev Rheumatol. 6, 683–692 (2010). 21060334 10.1038/nrrheum.2010.176 PMC3135416

[b6] Watson C. T., Disanto G., Breden F., Giovannoni G. & Ramagopalan S. V. Estimating the proportion of variation in susceptibility to multiple sclerosis captured by common SNPs. Sci Rep. 2, 770 (2012). 23105968 10.1038/srep00770 PMC3480808

[b7] Wang C. M. *et al.* Genetic variations in Toll-like receptors (TLRs 3/7/8) are associated with systemic lupus erythematosus in a Taiwanese population. Sci Rep. 4, 3792. (2014). 24445780 10.1038/srep03792 PMC3896912

[b8] Liu Y., Jones B., Brady W., Janeway C. A. Jr & Linsley P. S. Co-stimulation of murine CD4 T cell growth: cooperation between B7 and heat-stable antigen. Eur J Immunol. 22, 2855–2859 (1992). 1385153 10.1002/eji.1830221115

[b9] Nielsen P. J. *et al.* Altered erythrocytes and a leaky block in B-cell development in CD24/HSA-deficient Mice. Blood. 89, 1058–1067 (1997). 9028339

[b10] Bai X. F. *et al.* CD24 Controls expansion and persistence of autoreactive T Cells in the central nervous system during experimental autoimmune encephalomyelitis. J Exp Med. 200, 447–458 (2004). 15314074 10.1084/jem.20040131 PMC2211938

[b11] Ahmed M. A. *et al.* CD24 shows early upregulation and nuclear expression but is not a prognostic marker in colorectal cancer. J Clin Pathol. 62, 1117–1122 (2009). 19946098 10.1136/jcp.2009.069310

[b12] Zhang X. *et al.* CD24 on thymic APCs regulates negative selection of myelin antigen-specific T lymphocytes. Eur J Immunol. 42, 924–935 (2012). 22213356 10.1002/eji.201142024 PMC3359065

[b13] Liu Y., Wenger R. H., Zhao M. & Nielsen P. J. Distinct costimulatory molecules are required for the induction of effector and memory cytotoxic T lymphocytes. J Exp Med. 185, 251–262 (1997). 9016874 10.1084/jem.185.2.251 PMC2196124

[b14] Hough M. R. *et al.* Reduction of early B lymphocyte precursors in transgenic mice overexpressing the murine heat-stable antigen. J Immunol. 156, 479–488 (1996). 8543797

[b15] Hahne M., Wenger R. H., Vestweber D. & Nielsen P. J. The heat-stable antigen can alter very late antigen 4-mediated adhesion. J Exp Med. 179, 1391–1395 (1994). 8145052 10.1084/jem.179.4.1391 PMC2191441

[b16] Zarn J. A. *et al.* The small cell lung cancer antigen cluster-4 and the leukocyte antigen CD24 are allelic isoforms of the same gene (CD24) on chromosome band 6q21. Cytogenet Cell Genet. 70, 119–125 (1995). 7736776 10.1159/000134075

[b17] Zhou Q. *et al.* CD24 is a genetic modifier for risk and progression of multiple sclerosis. Proc Natl Acad Sci U S A. 100, 15041–15046 (2003). 14657362 10.1073/pnas.2533866100 PMC299898

[b18] Cui Y. Z. *et al.* Study on the association between CD24 gene polymorphisms and multiple sclerosis. Zhongfeng Yu Shen Jing Ji Bing Za Zhi 23, 155–157 (2006). Chinese.

[b19] Otaegui D. *et al.* CD24 V/V is an allele associated with the risk of developing multiple sclerosis in the Spanish population. Mult Scler. 12, 511–514 (2006). 16900767 10.1191/135248506ms1314sr

[b20] Sánchez E. *et al.* Association of a CD24 gene polymorphism with susceptibility to systemic lupus erythematosus. Arthritis Rheum. 56, 3080–3086 (2007). 17763438 10.1002/art.22871

[b21] Ronaghi M., Vallian S. & Etemadifar M. CD24 gene polymorphism is associated with the disease progression and susceptibility to multiple sclerosis in the Iranian population. Psychiatry Res. 170, 271–272 (2009). 19896210 10.1016/j.psychres.2009.01.002

[b22] Piotrowski P., Lianeri M., Wudarski M., Łacki J. K. & Jagodziński P. P. CD24 Ala57Val gene polymorphism and the risk of systemic lupus erythematosus. Tissue Antigens. 75, 696–700 (2010). 20230526 10.1111/j.1399-0039.2010.01447.x

[b23] González S. J. *et al.* CD24 as a genetic modifier of disease progression in multiple sclerosisin Argentinean patients. J Neurol Sci. 307, 18–21 (2011). 21641619 10.1016/j.jns.2011.05.032

[b24] Kollaee A., Ghaffarpor M., Pourmahmoudian H., Shahbazi M. & Zamani M. Investigation of CD24 and its expression in Iranian relapsing-remittingmultiple sclerosis. Int J Neurosci. 121, 684–690 (2011). 21815873 10.3109/00207454.2011.610529

[b25] Gao H., Song J. Q. & Wang J. Association of CD24 gene polymorphisms with systemic lupus erythematosus. Wuhan Da Xue Xue Bao (Yi Xue Ban) 35, 765–768 (2014). Chinese.

[b26] Thakkinstian A., McElduff P., D'Este C., Duffy D. & Attia J. A method for meta-analysis of molecular association studies. Stat Med. 24, 1291–1306 (2005). 15568190 10.1002/sim.2010

[b27] DerSimonian R. & Laird N. Meta-analysis in clinical trials. Control Clin Trials. 7, 177–188 (1986). 3802833 10.1016/0197-2456(86)90046-2

[b28] Mantel N. & Haenszel W. Statistical aspects of the analysis of data from retrospective studies of disease. J Natl Cancer Inst. 22, 719–748 (1959). 13655060

[b29] McDonald W. I. *et al.* Recommended diagnostic criteria for multiple sclerosis: guidelines from theInternational Panel on the diagnosis of multiple sclerosis. Ann Neurol. 50, 121–127 (2001). 11456302 10.1002/ana.1032

[b30] Poser C. M. *et al.* New diagnostic criteria for multiple sclerosis: guidelines for research protocols. Ann Neurol. 13, 227–231 (1983). 6847134 10.1002/ana.410130302

[b31] Hochberg M. C. Updating the American college of rheumatology revised criteria for the classification of systemic lupus erythematosus. Arthritis Rheum. 40, 1725 (1997). 10.1002/art.1780400928 9324032

[b32] Lu X. *et al.* Contribution of NKX2-3 polymorphisms to inflammatory bowel diseases: a meta-analysis of 35358 subjects. Sci Rep. 4, 3924 (2014). 24473197 10.1038/srep03924 PMC5379238

[b33] Liu Y. & Zheng P. CD24: a genetic checkpoint in T cell homeostasis and autoimmune diseases. Trends Immunol. 28, 315–320 (2007). 17531534 10.1016/j.it.2007.05.001

[b34] Suzuki T. *et al.* CD24 induces apoptosis in human B cells via the glycolipid-enriched membrane domains/rafts-mediated signaling system. J Immunol. 166, 5567–5577 (2001). 11313396 10.4049/jimmunol.166.9.5567

[b35] Bai X. F. *et al.* The heat-stable antigen determines pathogenicity of self-reactive T cells in experimental autoimmune encephalomyelitis. J Clin Invest. 105, 1227–1232 (2000). 10791997 10.1172/JCI9012 PMC315444

[b36] Liu J. Q. *et al.* CD24 on the resident cells of the central nervous system enhances experimental autoimmune encephalomyelitis. J Immunol. 178, 6227–6235 (2007). 17475850 10.4049/jimmunol.178.10.6227

[b37] Carl J. W. Jr *et al.* Autoreactive T cells escape clonal deletion in the thymus by a CD24-dependent pathway. J Immunol. 181, 320–328 (2008). 18566397 10.4049/jimmunol.181.1.320

[b38] Wither J. E., Paterson A. D. & Vukusic B. Genetic dissection of B cell traits in New Zealand black mice. The expanded population of B cells expressing up-regulated costimulatory molecules shows linkage to Nba2. Eur J Immunol. 30, 356–365 (2000). 10671190 10.1002/1521-4141(200002)30:2<356::AID-IMMU356>3.0.CO;2-H

