# Supplementary material for: Association between the CD24 Ala57Val polymorphism and risk for multiple sclerosis and systemic lupus erythematosus: a meta-analysis
Source: Sci Rep. 2015 Apr 1;5:9557. doi: 10.1038/srep09557 (PMC5381688; doi:10.1038/srep09557)
Supplement: Supplementary Information [file srep09557-s1.pdf]

Association between the *CD24* Ala57Val polymorphism and risk for multiple sclerosis and systemic lupus erythematosus: a meta-analysis

Jian Huang*, Yaqi Yang*, Zibin Liang, Miaomiao Kang, Ying Kuang, Feng Li

Department of Anatomy and Neurobiology, Zhongshan School of Medicine, Sun Yat-sen University, Guangzhou, China

Correspondence to: Prof. Feng Li, Department of Anatomy and Neurobiology, Zhongshan School of Medicine, Sun Yat-sen University, Guangzhou 510080, China

Tel: +86-20-87332198

Email: lifeng@mail.sysu.edu.cn

*These authors contributed equally to this work.

Supplementary Table 1. Scale for methodological quality assessment.

**Criteria**	**Score**
Selection	
Is the case definition adequate	1
Representativeness of the cases	1
Selection of controls	1
Definition of controls	1
Comparability	
Comparability of cases and controls on the basis of the design or analysis	2
Exposure	
Ascertainment of exposure	1
Same method of ascertainment for cases and controls	1
Non-response rate	1

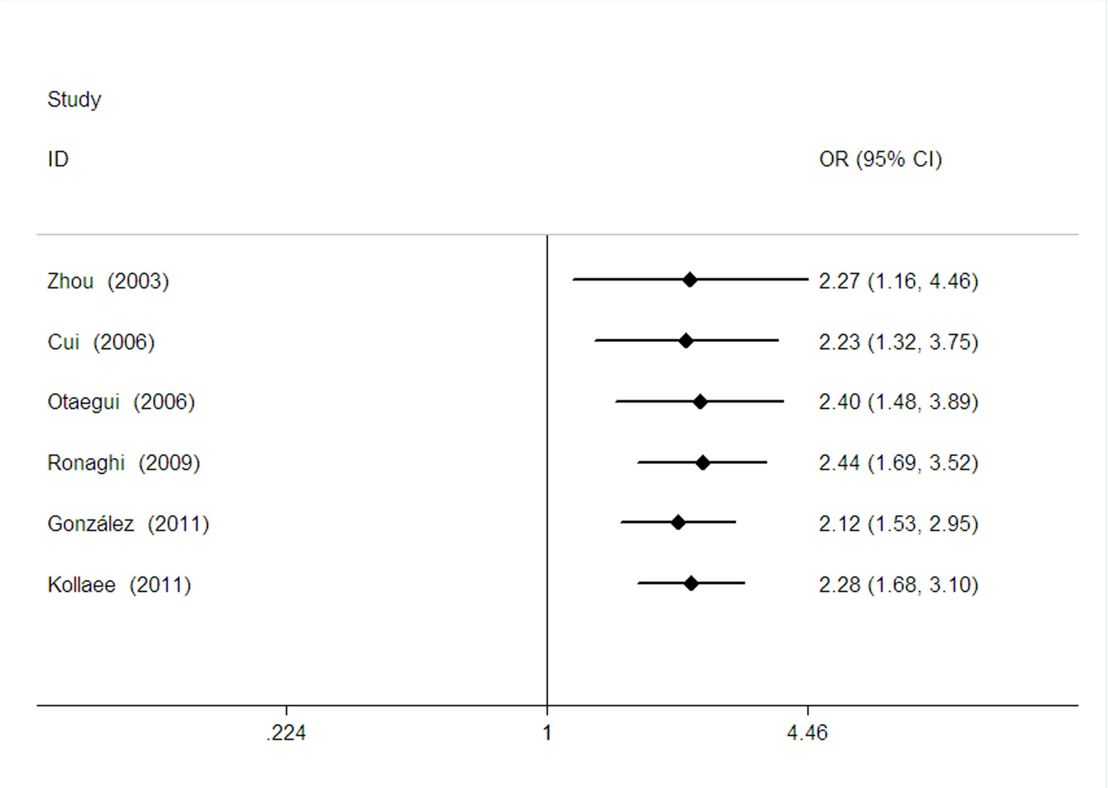

Supplementary Figure 1 Cumulative meta-analysis of the *CD24* Ala57Val polymorphism and MS risk.

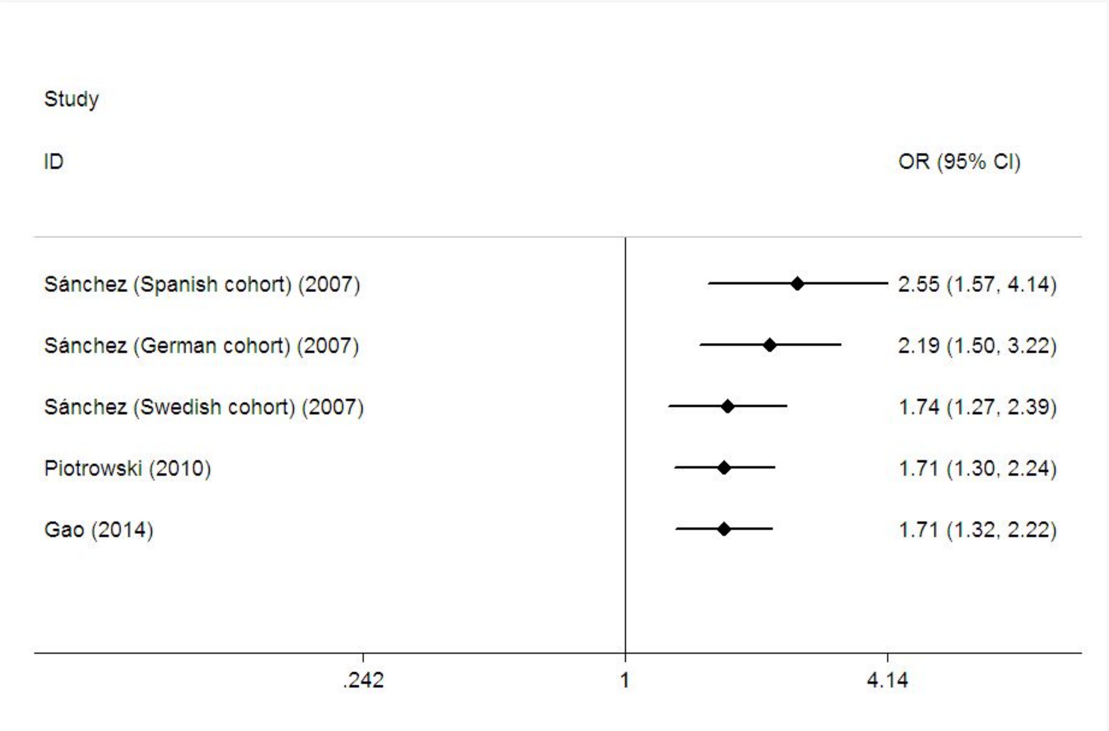

Supplementary Figure 2 Cumulative meta-analysis of the *CD24* Ala57Val polymorphism and SLE risk.
